# Supplementary material for: Critical Evaluation of Sinonasal Disease in 64 Adults with Primary Ciliary Dyskinesia
Source: J Clin Med. 2019 May 7;8(5):619. doi: 10.3390/jcm8050619 (PMC6571605; doi:10.3390/jcm8050619)
Supplement: Supplementary file 1 [file jcm-08-00619-s001.pdf]

Journal of
Clinical Medicine

MDPI

Table S1: PCD diagnostic tests

	mean	(± SD)
Age at diagnosis of patients with SI and/or respiratory distress (years)	16.5	15
Age at diagnosis of other patients (years)	30	14
	Patient (n)	%
(NO) (nL/min) (*n* = 45)*		
n(NO) <77 nL/min	38	84.4
n(NO) ≥77 nL/min	7	15.6
Ciliary beat frequency (CBF) (*n* = 59)**		
Complete immotility	40	67.9
Low CBF <8Hz	10	17
CBF>8Hz	8	13.3
Not evaluable	1	1.8
Ciliary ultrastructure (*n* = 62)		
Absence of outer dynein arms	17	27.4
Absence of both dynein arms	14	22.6
Central complex abnormalities	14	22.6
Absence of inner dynein arms with disorganisation	11	17.7
No detectable defect***	6	9.7
Causal biallelic mutation	50	77

* Cut- off determined by Jackson CL, Behan L, Collins SA, et al. Accuracy of diagnostic testing in primary ciliary dyskinesia. The European Respiratory Journal. 2016;47(3):837-848.

** During the20 year'study, technical modifications were introduced for the evaluation of ciliary beat frequency measurements (Papon J-F, Bassinet L, Cariou-Patron G, Zerah-Lancner F, Vojtek A-M, Blanchon S, et al. Quantitative analysis of ciliary beating in primary ciliary dyskinesia: A pilot study. Orphanet J Rare Dis. 2012;7:78). We considered 3 groups: Dyskinetic cilia with low CBF (<8Hz), dyskinetic cilia with normal CBF (>8Hz), or complete immotility when almost all cilia are immotile

*** All patients presented Kartagener syndrome

Abbreviations SI = Situs Inversus, SD = Standard Deviation, n(NO) = Nasal nitric oxide, CBF = Ciliary Beat Frequency
